# Editorial: Forces in Biology - Cell and Developmental Mechanobiology and Its Implications in Disease

**DOI:** 10.3389/fcell.2020.598179

**Published:** 2020-09-30

**Authors:** Selwin K. Wu, Guillermo A. Gomez, Samantha J. Stehbens, Michael Smutny

**Affiliations:** ^1^Department of Cell Biology, Harvard Medical School, Boston, MA, United States; ^2^Department of Pediatric Oncology, Dana-Farber Cancer Institute, Boston, MA, United States; ^3^Centre for Cancer Biology, SA Pathology and University of South Australia, Adelaide, SA, Australia; ^4^Cell Biology and Molecular Medicine Division, Institute for Molecular Bioscience, University of Queensland, Brisbane, QLD, Australia; ^5^Centre for Mechanochemical Cell Biology & Warwick Medical School, University of Warwick, Coventry, United Kingdom

**Keywords:** mechanobiology, cell biology, developmental biology, cardiovascular, signaling, stem cells, neuroscience, bone

Mechanobiology despite widely regarded as an emerging discipline, its concept, is proposed more than a hundred years ago by mathematical biologist D'Arcy Wentworth Thompson. In his 1917's book *On Growth and Form* (Thompson, 1992), life forms were envisioned to reflect physics and mathematical principles. Since then mechanobiologists endeavored to transform cell and developmental biology into a field of quantitative science and technology, accelerated by the development and application of new approaches including virtual power theory (Putra et al.) and random positioning machines (Bradbury et al.).

In the past three decades, the authoritative concept that “phenotype can dominate over genotype” (Nelson and Bissell, 2006) by Mina Bissell and others have further underscored the overarching role of tissue and cellular mechanisms in health and disease. Indeed, an increase of matrix stiffness can promote tissue transformation through abnormal mitosis and signaling from adhesion receptors (Butcher et al., 2009; Knouse et al., 2018). Nevertheless, the quantitative nature of sequence analysis in the field of genomics have almost realized the promise of personalized medicine. In this post-genomic era, we envision that cell, developmental mechanobiology and its pathogenesis could, in principle, drive a new form of personalized medicine; that would emerge from the quantitative description of molecular, cell, and tissue dynamics (Putra et al.). In this issue, we highlight recent developments in six chapters: namely (1) stem cells mechanobiology, (2) bone biomechanics & cellular physiology, (3) Rho GTPases signaling, (4) cardiovascular mechanobiology, (5) mechanobiology of the brain, and (6) molecular mechanisms of mechanobiology.

## Chapter 1: Stem Cells Mechanobiology

Stem cells and their local microenvironment communicate via mechanical cues to regulate gene expression that influence developmental processes. Domingues et al. showed that Cofilin-1 is a mechanosensitive regulator of transcription in human umbilical cord mesenchymal stem/stromal cells. Cofilin-1 activity is regulated by substrate stiffness and activated cofilin-1 promoted transcription in human umbilical cord mesenchymal stem/stromal cells (Domingues et al.).

With regards to stem cell-based treatment, pluripotent stem cells can revolutionize regenerative medicine as they have the potential to form organs. However, the scaling up of pluripotent stem cells expansion is required to achieve the goal of therapeutic use. Chan et al. reviewed recent advances in pluripotent stem cell expansion focusing on *in vitro* design of two-dimensional surface modification and three-dimensional encapsulation, which can provide the mechanical cues required for the expansion of pluripotent stem cells.

Besides regenerative medicine, the integration of big data obtained from mechanobiology and stem cell studies remains a challenge. To that end Putra et al. summarize various approaches, including a perfusion chamber platform, to analyse biomechanics. They proposed the study of mechanobiology at the “omics” level, thus termed mechanomics. To integrate “omics” data, various computational modeling approaches are applied for the mapping of the mechanome of biological systems including stem cells, bones and tissues (Putra et al.).

## Chapter 2: Bone Biomechanics and Cellular Physiology

The osteochondral interface between the bone and cartilage facilitates signaling crosstalk and nutritional molecules exchange, thus enabling an integrated response to mechanical stimuli. Oliveira Silva et al. describe the molecular interactions and transport in the osteochondral interface and discuss the potential of using contrast agents for studying molecular transport and structural changes of the joint.

Cellular physiology and our bones are profoundly influenced by gravitational forces. Bradbury et al. reviewed the impact of simulated microgravity on the cytoskeleton, extracellular matrix synthesis and bone cell signaling.

## Chapter 3: Rho GTPases Signaling

GTPase signaling is an important aspect of mechanobiology. In particular, RhoGTPases are canonical upstream regulators of actomyosin structure and function controlling cellular mechanics (Wu and Priya). Feedback signaling pathways are found to complete the RhoGTPases signaling loop. Myosin II controls RhoGTPases signaling through multiple mechanisms, namely contractility driven advection (Moore et al., 2014), scaffolding (Priya et al., 2015), and sequestration of signaling molecules. Wu and Priya discuss these mechanisms by which myosin II regulates RhoGTPase signaling in cell adhesion, migration, and tissue morphogenesis.

The genetic regulation of Rho GTPases may also play an important role in pathology such as recurrent miscarriage. The small G-protein Rnd3 (or RhoE) is implicated as a therapeutic target in recurrent miscarriage. Ma et al. suggest that the upregulation of Rnd3 by forkhead box D3 (FOXD3), a key transcription factor that binds to the RND3 core promoter region, regulated trophoblast migration and proliferation via the RhoA-ROCK1 signaling pathway and inhibits apoptosis via ERK1/2 signaling.

## Chapter 4: Cardiovascular Mechanobiology

Forces emerge from and act on the cardiovascular system during development and from the heart pumping of blood flow. Simpson et al. reviewed the role of biomechanical stress-induced translational control in the heart and vascular cells. Several specific areas were reviewed including protein translation regulation by mechanical stimuli, mTOR signaling, endoplasmic reticulum stress, atherosclerosis, and cardiac hypertrophy.

Next, Gaetani et al. reviewed a combination of theoretical models of cell and tissue mechanics and experimental findings. Tissue engineering and induced pluripotent stem cell (iPSC) technologies were then discussed as potential personalized medicine strategies to treat heart failure.

## Chapter 5: Mechanobiology of the Brain

Besides the cardiovascular system, the neuronal system is another exciting area covered in this issue including cell interactions and migration of neurons. Veeraval et al. reviewed the role of adherens junctions in cortical brain development. The reciprocal interactions between adherens junctions and the epithelial polarity complexes that promote glial apicobasal polarity are highlighted. Several key processes of cortical brain development are integrated including asymmetric cell division, with a focus on the mechanisms that integrate adherens junctions and the actin cytoskeleton (Wu and Yap, 2013; Wu et al., 2014). Processes underlying cortical malformations are also discussed.

In the brain, neuronal migration is a critical step during the formation of functional neural circuits. Nakazawa and Kengaku reviewed the synergistic role of actomyosin microfilaments and microtubule motor proteins, in generating mechanical forces to translocate the nuclei of newborn neurons through confined space. In addition, the authors concluded that the mechanical properties of the nucleus and surrounding tissues not only contribute to force transmission but also present a physical barrier to nuclear translocation.

## Chapter 6: Molecular Mechanisms of Mechanobiology

Following up on cytoskeletal force exertion on the nucleus. Terriac et al. describe and measure the turnover of vimentin intermediate filament rings that deform the nucleus during cell spreading in cells. Indeed, force exertion on nucleus can often lead to transcriptional changes. Accordingly, Martinez et al. highlighted the regulation of Yap/Taz nuclear translocation, transcription and mechanotransduction with the use of atomic force microscopy.

Lastly, with a focus on the epithelium of mammary gland, pancreas, and urinary system. Stewart and Davis review the role of the mechanosensitive ion channel PIEZO1 in the development, function, and dysfunction of epithelia.

In this issue, we presented a broad spectrum of work from fundamental cell biology to articles at the interface of development and disease. We believe that this issue will be of interest to students and researchers studying the molecular and cellular mechanisms in development and diseases, enabling them to appreciate how clearer understanding of these mechanisms can inspire therapeutics and diagnostics.

## Author Contributions

SW wrote the manuscript. GG edited the manuscript. All authors provided intellectual input to the editorial.

## Conflict of Interest

The authors declare that the research was conducted in the absence of any commercial or financial relationships that could be construed as a potential conflict of interest.
